# Genome-wide identification of accessible chromatin regions by ATAC-seq upon induction of the transcription factor bZIP11 in Arabidopsis

**DOI:** 10.1038/s41597-023-02395-6

**Published:** 2023-07-27

**Authors:** Alicia M. Hellens, Jazmine L. Humphreys, Franziska Fichtner, Miloš Tanurdžić, Christine A. Beveridge, François F. Barbier

**Affiliations:** 1 School of Biological Sciences, The University of Queensland, St Lucia, QLD 4072 Australia; 2 ARC Centre for Plant Success in Nature and Agriculture, The University of Queensland, St Lucia, QLD 4072 Australia; 3 School of Natural Sciences, University of Tasmania, Hobart, Tasmania Australia; 4 Institute for Plant Biochemistry, Heinrich Heine University, Dusseldorf, Germany

**Keywords:** Chromatin remodelling, Plant molecular biology

## Abstract

Basic leucine zipper 11 (bZIP11) is a transcription factor that is activated under low energy conditions in plants and plays a crucial role in enabling plants to adapt to starvation situations. Although previous results indicate that bZIP11 regulates chromatin accessibility based on evidence obtained from single genomic loci, to what extent this transcription factor regulates the chromatin landscape at the whole genome level remains unknown. Here we addressed this by performing an ATAC-seq (Assay for Transposase-Accessible Chromatin with high-throughput sequencing) on *Arabidopsis thaliana* (Arabidopsis) leaf protoplasts to obtain a profile of chromatin patterning in response upon bZIP11 induction. We identified, on average, 10,000 differentially accessible regions upon bZIP11 induction, corresponding to over 8,420 different genes out of the 25,000 genes present in the Arabidopsis genome. Our study provides a resource for understanding how bZIP11 regulates the genome at the chromatin level and provides an example of the impact of a single transcription factor on a whole plant genome.

## Background & Summary

Basic leucine zipper11 (bZIP11) is a transcription factor (TF) which regulates gene expression during low-energy conditions in plants and enables plants to adjust their metabolism, growth, and development to such unfavourable conditions^1–3^. In *Arabidopsis thaliana* (Arabidopsis), bZIP11 belongs to a group of five proteins involved in sugar responses, named the S_1_ bZIP group^2,4–8^. It is estimated by DNA Affinity Purification with high throughput sequencing (DAP-seq) that bZIP11 contains DNA-binding sites in over 7,000 genes in Arabidopsis, which is nearly one third of the entire genes in the genome^9^. The bZIP TFs bind DNA at *cis*-regulatory elements (CREs) known as G-boxes, which all have a conserved ACGT core flanked by cytosines or guanines (C/GACGTG/C)^10^. bZIP11 has been demonstrated to promote the expression of specific auxin-related genes by recruiting histone acetylation machinery to enhance chromatin accessibility^3,11^. However, the extent to which bZIP11 impacts chromatin accessibility at the whole-genome scale remains unknown.

Assay for Transposase-Accessible Chromatin with high throughput sequencing (ATAC-seq) is a technique which can determine genome-wide locations of chromatin accessible to transposase insertion and by extension, other proteins, for example TFs^12,13^. This technique is rapid and can be performed on small quantities of starting material. Previous studies have performed ATAC-seq on different plant organs, conditions, or in response to different treatments to determine changes in accessibility of those DNA regions that are likely to contain regulatory sequences^14–17^. Here we report ATAC-seq results in response to induction of a single plant TF, with known regulatory sequences, to determine the genome-wide effect of this TF on chromatin accessibility in Arabidopsis.

**Fig. 1:**
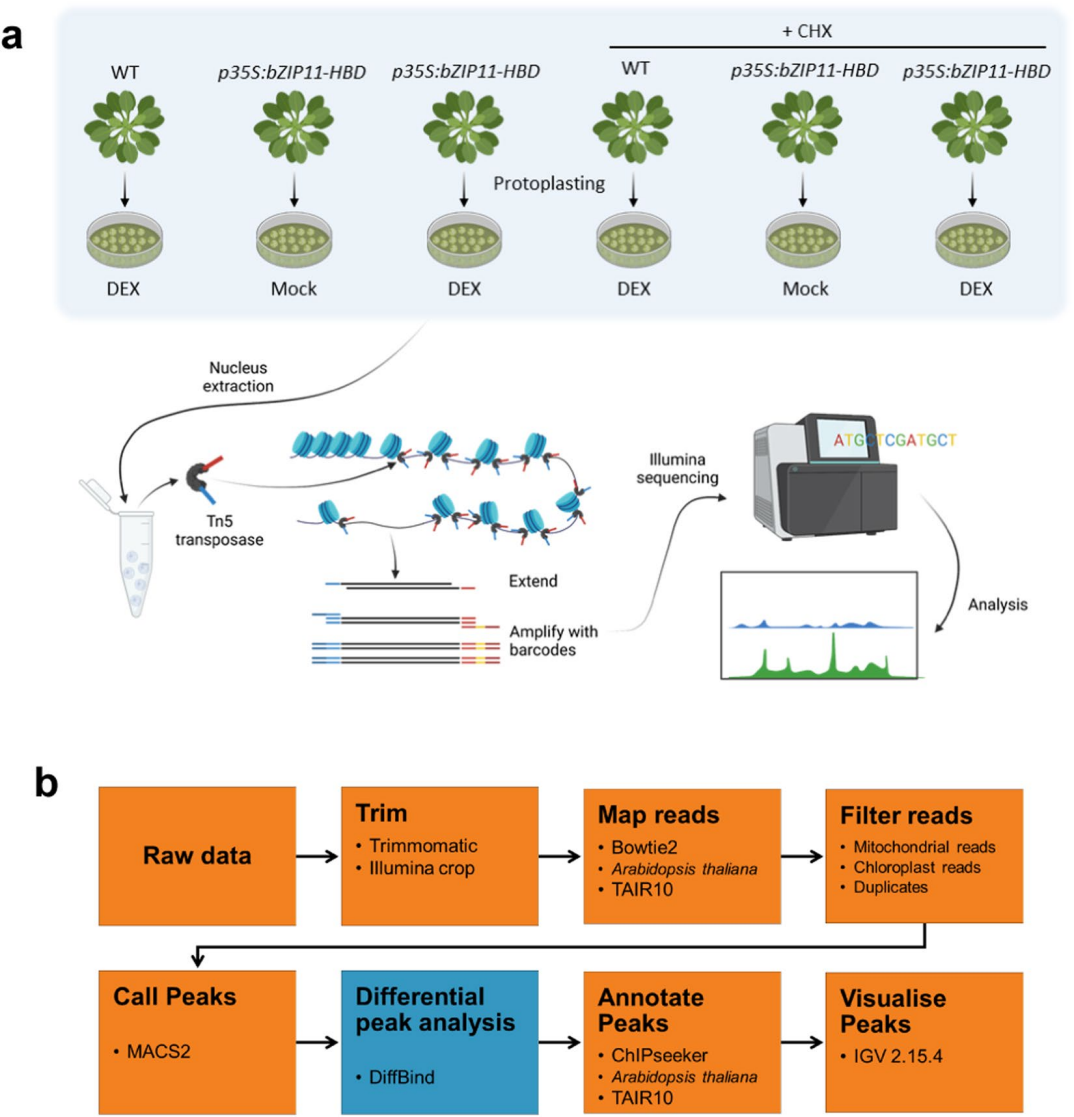
Overview of experimental design and data analysis workflow. (**a**) Experimental design and ATAC-seq library preparation. Arabidopsis protoplasts of four-week-old wild type (Col-0) or *p35S:bZIP11-HBD* were isolated. Protoplasts were then treated with different combinations of 10 µM dexamethasone, acetone, water, and 35 µM of cycloheximide (CHX). Each condition was carried out in four biological replicates. After nucleus extraction, samples were incubated with a hyperactive Tn5 transposase which adds adapters onto the DNA. DNA regions were amplified using primers against the adapters, so only accessible regions are amplified. PCR also added sequencing barcodes allowing for samples to be pooled and sent for sequencing. (**a**) was adapted from Buenrostro *et al*., 2015. (**b**) The analysis workflow of ATAC-seq orange boxes indicate step was carried out using Galaxy Australia. Blue indicates step was carried out in R.

To achieve a comprehensive profile of accessible chromatin regions and discover new downstream targets of bZIP11, we generated 24 chromatin accessibility data sets from wild-type (WT) Arabidopsis protoplast samples, as well as from transgenic *p35S:bZIP11-HBD* Arabidopsis plants^18^. In these *p35S:bZIP11-HBD* plants, the expression of *bZIP11* coding sequence is driven by the cauliflower mosaic virus, *CaMV35S*, promoter (*p35S*) that leads to ectopic gene expression in the whole plant. A rat glucocorticoid receptor, or hormone binding domain (HBD), is fused to bZIP11, and retains this TF in the cytoplasm. Upon treatment with dexamethasone (DEX), which binds to the HBD, the translocation of bZIP11 to the nucleus is enabled, allowing its TF activity to occur^19^. Four-week-old Arabidopsis plants were used to extract leaf protoplasts from approximately six leaves per plant (experiment summarised in Fig. 1a). Protoplasts were treated for 45 minutes after a one hr recovery period following protoplast extraction. Four biological replicates were treated with either DEX, which induces the translocation of bZIP11 protein into the nucleus, or acetone, the solvent for DEX (Mock control). Using protoplasts allows for a rapid and homogeneous chemical uptake which reduces variability between samples^20^. In parallel, the samples were treated, prior to DEX or acetone treatment, with cycloheximide (CHX), a protein biosynthesis inhibitor^21^ that enables to discriminate the direct targets of bZIP11 from its indirect targets. Approximately 50,000 cells from each replicate of each treatment were used for ATAC-seq. In total, approximately 10,000 regions of the genome were determined as differentially accessible regions (DARs) upon induction of bZIP11 translocation to the nucleus, depending on which control was used (Table 1). These DARs are summarised by a core list of 2,553 genes identified to have their chromatin accessibility directly influenced by bZIP11.

**Table 1:** Summary of ATAC-seq sequencing, mapping, and peak calling.

Genotype	Treatment	Sample ID	Raw Reads	Clean Reads	Mapped Reads	Called Peaks	Accession	DiffBound Peaks
Col -0	Dex	AH025	14,017,763	11,559,584	7,335,921	19,874	SAMN34226419	10,140 (8,843 genes)
Col -0	Dex	AH028	10,143,097	7,007,692	4,925,970	18,618	SAMN34226420	
Col -0	Dex	AH027	11,313,278	7,305,956	4,891,108	19,857	SAMN34226421	
Col -0	Dex	AH028	9,972,594	6,476,678	4,631,287	19,814	SAMN34226422	
35 S:bZIP11 -HBD	Mock	AH029	23,738,704	8,984,170	6,593,699	19,301	SAMN34226423	11,236 (9,664 genes 3339 gained 2518 lost)
35 S:bZIP11 -HBD	Mock	AH030	23,487,379	19,317,810	11,256,808	21,167	SAMN34226424	
35 S:bZIP11 -HBD	Mock	AH031	11,564,134	8,689,856	6,144,551	20,210	SAMN34226425	
35 S:bZIP11 -HBD	Mock	AH032	10,904,533	5,642,758	4,334,768	19,474	SAMN34226426	
35 S:bZIP11 -HBD	Dex	AH033	51,908,821	31,990,286	16,607,717	21,979	SAMN34226427	
35 S:bZIP11 -HBD	Dex	AH034	22,101,174	21,768,988	12,416,405	22,757	SAMN34226428	
35 S:bZIP11 -HBD	Dex	AH035	26,803,740	25,482,872	14,052,875	22,081	SAMN34226429	
35 S:bZIP11 -HBD	Dex	AH036	26,846,853	24,840,140	13,471,482	22,638	SAMN34226430	
Col - 0	CHX + dex	AH037	8,242,523	5,100,362	3,816,207	18,200	SAMN34226431	8,154 (6,689 genes)
Col - 0	CHX + dex	AH038	9,959,240	6,938,240	4,974,236	18,479	SAMN34226432	
Col - 0	CHX + dex	AH039	22,104,359	11,316,082	7,326,410	21,035	SAMN34226433	
Col - 0	CHX + dex	AH040	20,066,853	11,752,986	7,625,434	20,872	SAMN34226434	
35 S:bZIP11 -HBD	CHX + mock	AH041	11,576,489	7,333,816	5,443,565	19,553	SAMN34226435	11,483 (8,967 genes 2,021 lost 4,319 gained)
35 S:bZIP11 -HBD	CHX + mock	AH042	9,287,775	5,451,356	4,190,938	17,891	SAMN34226436	
35 S:bZIP11 -HBD	CHX + mock	AH043	13,856,888	8,288,610	6,010,492	20,168	SAMN34226437	
35 S:bZIP11 -HBD	CHX + mock	AH044	7,820,098	6,459,694	4,927,170	18,343	SAMN34226438	
35 S:bZIP11 -HBD	CHX + dex	AH045	32,518,073	15,864,764	9,527,833	22,393	SAMN34226439	
35 S:bZIP11 -HBD	CHX + dex	AH046	32,036,847	28,842,408	15,347,729	22,072	SAMN34226440	
35 S:bZIP11 -HBD	CHX + dex	AH047	15,953,569	13,695,372	8,699,347	21,386	SAMN34226441	
35 S:bZIP11 -HBD	CHX + dex	AH048	23,280,510	20,464,908	11,939,899	22,177	SAMN34226442	

DiffBound peaks were determined by comparing bZIP11-induced samples with each control (WT + Dex treated and bZIP11-inducibe + Mock treated) for both with and without CHX.

## Methods

### Plant materials

*Arabidopsis thaliana* seeds were stratified for three days at four degrees Celsius then transferred to growth chambers with 16 h light: 8 h dark, 22 °C day: 20 °C night with 150 ± 20 µmol m^−2^ s^−1^ light intensity. Plants were grown in UQ23 potting mix (70% composted pine bark (zero to five mm), 30% cocopeat, supplemented with dolomite and osmocote).

### Protoplast isolation

Four-week-old wild type (WT) Columbia-0 (Col-0) and inducible bZIP11 (*p35S:bZIP11-HBD*)^18^ plants were used to extract mesophyll protoplasts via the epidermal leaf peel method^22^. Six leaves were placed in 10 mL of enzyme solution (1% Cellulase ‘Onozuka’ R10, 0.25% maceroenzyme ‘Onozuka’ R10, 0.4 M mannitol, 20 mM KCl, 20 mM MES, 10 mM CaCl2, 0.1% BSA, adjust to pH 5.7) and digested for 1 h with constant gentle agitation. Cells were filtered through 50 μM mesh (CellTrics, Sysmex, Norderstedt, Germany) then washed twice in W5 (154 mM NaCl, 125 mM CaCl_2_, 5 mM KCl, 2 mM MES). Protoplasts were then re-suspended in MMg (0.4 M mannitol, 15 mM MgCl_2_, 4 mM MES, adjust to pH 5.7) at a concentration of 200,000 cells per mL. Reactions took place in 2 mL, in six-well plastic plates with constant agitation. WT cells were treated with 10 μM DEX, Mock plants were treated with acetone, and bZIP11-inducible plants were treated with 10 μM DEX. For CHX-treated reactions, CHX was added prior to DEX or acetone treatment at a concentration of 35 μM. 45 min after treatment, cells were collected by centrifugation and resuspended at a concentration of approximately 50,000 cells per sample.

### RNA extraction and gene expression

Total RNA was extracted (NucleoSpin RNA Extraction, MACHEREY-NAGEL, Düren, Germany), reverse transcribed into cDNA (iScript Supermix, Bio-Rad Laboratories, California, USA), and diluted cDNA was used as a template for quantitative Real-Time PCR (SensiFASTTM SYBR® No-ROX Kit from Bioline) according to manufacturer’s instructions. Fluorescence was measured using a CFX384 TouchTM Real-Time PCR Detection System (Bio-Rad Laboratories, California, USA) using the following protocol: 95 °C for 3 min, 40 cycles at 10 s each for 95 °C and 59 °C for 45 s, and 1 min each for 95 °C and 55 °C. The ΔΔCt technique was used to determine gene expression and primer efficiency was used to correct it. The geomean expression of two technical replicates of each, *TUBULIN3* (At5g62700), *ACTIN* (Combination of *ACT2*, *ACT7*, and *ACT8*: At3Gg18780, At5g09810, and At1g49240), and *18 S* (18 S rRNA) were used to normalise gene expression. Every primer sequence used in this study is outlined in Supplementary Table 1.

### ATAC-seq protocol

ATAC-seq library preparation was performed as modified from Buenrostro *et al*.^12,13,23^. Following protoplast extraction, nuclei were isolated from approximately 50,000 cells per reaction by sucrose sedimentation, using a method modified from Bajic *et al*.^14^. Freshly extracted cells were centrifuged at 500 × g at 4 °C. The following steps were all carried out on ice. Supernatant was discarded and pellet was resuspended in 1 mL of ice-cold nuclei purification buffer (20 mM MOPS, 40 mM NaCl, 90 mM KCl, 2 mM EDTA, 0.5 mM EGTA, 0.5 mM spermidine, 0.2 mM spermine 1 × protease inhibitors, adjust to pH 7). Cells were then filtered through 30 μM mesh (CellTrics, Sysmex, Norderstedt, Germany). Nuclei were then spun down at 1200 × g for 10 min at 4 °C and pellet was resuspended in 1 mL of ice-cold nuclei extraction buffer 2 (0.25 m sucrose, 10 mM Tris-HCl pH8, 10 mM MgCl, 1% Triton X-100, 1 × protease inhibitors). This step was repeated but this time pellet was resuspended in 300 μL of NPB and this resuspension of nuclei was carefully layered over 300 μL of ice-cold nuclei extraction buffer 3 (1.7 M sucrose, 10 mM Tris-HCl pH 8, 2 mM MgCl, 0.15% Triton X-100 1 × protease inhibitor). The two layers were then spun down at 300 × g for 20 min at 4 °C following which, supernatant was removed. Nuclei were resuspended in 50 μL of tagmentation reaction mix, as per manufacturer instructions (TDE1, Illumina) and incubated at 37 °C for 30 min with gentle agitation every 5 min. Reactions were purified following manufacturer’s instructions using a QIAGEN MinElute PCR purification kit (catalogue number 28004) and eluted in 11 μL of elution buffer. DNA was amplified by PCR using ATAC barcoded primers and NEB Next High-Fidelity PCR Master Mix (5 min 72 °C, 30 sec. 98 °C, then 5 × (10 sec. 98 °C, 30 sec. 63 °C, 1 min 72 °C) held at 4 °C). 5 μL of the PCR reaction was then further amplified by quantitative PCR (qPCR) (30 sec. 98 °C, then 20 × (10 sec. 98 °C, 30 sec. 63 °C, 1 min 72 °C)) to determine the required number of additional cycles. Additional cycle number for each reaction was determined by the cycle number for which a reaction has reached one third of its maximum, using the linear fluorescence vs cycle number graph from the qPCR. All libraries were purified with AMPure XP beads at a ratio of 1.5:1 beads:PCR reaction. Final elution in 20 μL of 10 mM Tris pH 8. Libraries were sequenced using Illumina HiSeq paired end 150 bp by NovoGene, Singapore.

### ATAC-seq data analysis

A summary of the ATAC-seq data analysis workflow used in this study is represented in Fig. 1b. Processing was carried out using Galaxy Australia^24^ and R with RStudio (Version 4.2.2) with the following steps. In Galaxy, raw reads were trimmed using Trimmomatic^25^ with the following settings: a 10 bp HEADCROP, a SLIDINGWINDOW with an average quality of 30 over every 6 bp, and an ILLUMINACLIP NexteraPE. Reads shorter than 30 bp and longer than 1000 bp were discarded. Reads were mapped against *Arabidopsis thaliana* TAIR10 reference genome (https://www.arabidopsis.org) using Bowtie2^26^, with paired end, dovetailing, and a maximum fragment length of 1000. Reads smaller than 30 bp, duplicate reads, reads with a quality score of <30 phred, and those which were mapped to the chloroplast or mitochondrial genome were discarded. Peaks were called with MACS2^27^, using the inputs: single-end BED, effective genome size 1.2e8, an extension size of 200 and a shift size of 100. BED and BAM and index files were then imported into RStudio and DARs were determined using the package DiffBind^28^. Peaks were read with peakCaller = “narrow”, minOverlap = 3 and dba.contrast function was specified to compare bZIP11-induced samples to either WT or Mock-treated samples. The package rtracklayer was used to convert the DARs determined by DiffBind into BED format. The peaks report was then imported back into Galaxy where differential peaks were annotated to the Arabidopsis reference genome using ChIPseeker^29^. The coordinates of peaks were compared with the annotated genome to determine the distribution of the peaks. ChIPseeker was also used to retrieve the nearest genes around differentially accessible peaks between the treatments. The resulting BED file of annotated DARs was imported into the Interactive Genome Viewer (Broad Institute, University of California)^30^ along with BED files DARs from MACS2 output for visualisation. STREME^31^ from the MEME suite was used to identify overrepresented motifs in the DAR sequences (minimum width = 4, maximum width = 15, p-value threshold = 0.05). Overrepresented motifs were then compared with DAP-seq motifs^9^ using TOMTOM^32^ to search for motifs overlapping by at least 5 bp.

## Data Records

ATAC-seq reads and peak files have been submitted to the National Center for Biotechnology Information Sequence Read Archive (NCBI SRA, PRJNA956597)^33^. https://trace.ncbi.nlm.nih.gov/Traces/?view=study&acc=SRP433061.

## Technical Validation

**Fig. 2:**
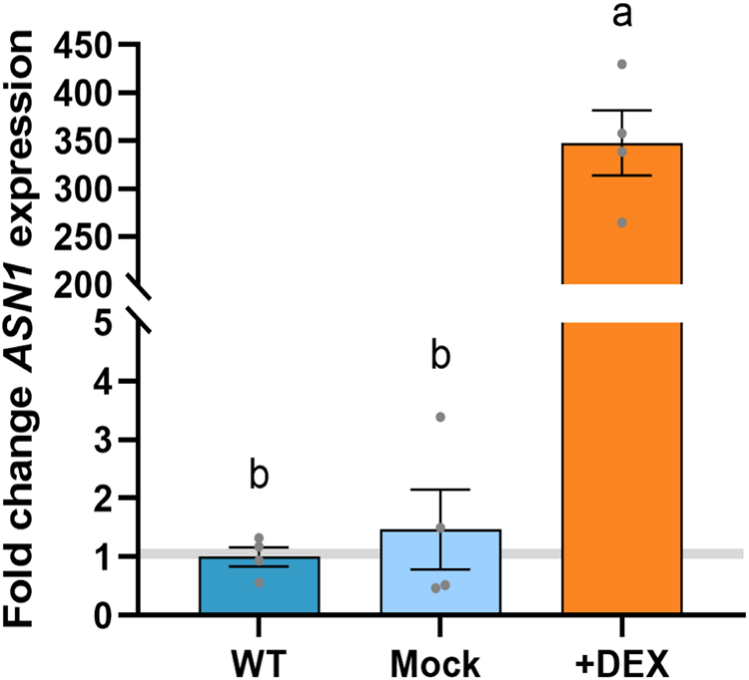
Validation of bZIP11 activity induction. Expression of the bZIP11 direct target *ASN1* in response to DEX treatment, Data are mean ± s.e.m (n = 4). Letters on the graph indicate statistical differences determined by one-way ANOVA using Tukey correction for multiple comparisons.

To validate the induction of bZIP11 activity upon DEX treatment, we measured the expression of *ASPARAGINE SYNTHASE1* (*ASN1*), a known direct target of this TF^18^. *ASN1* expression was nearly 350-fold upregulated in the DEX-treated *p35S:bZIP11-HBD* samples compared to the DEX-treated WT samples and the Mock-treated *p35S:bZIP11-HBD* samples (Fig. 2), confirming that DEX treatment efficiently induced bZIP11 activity in the *p35S:bZIP11-HBD* samples.

**Fig. 3:**
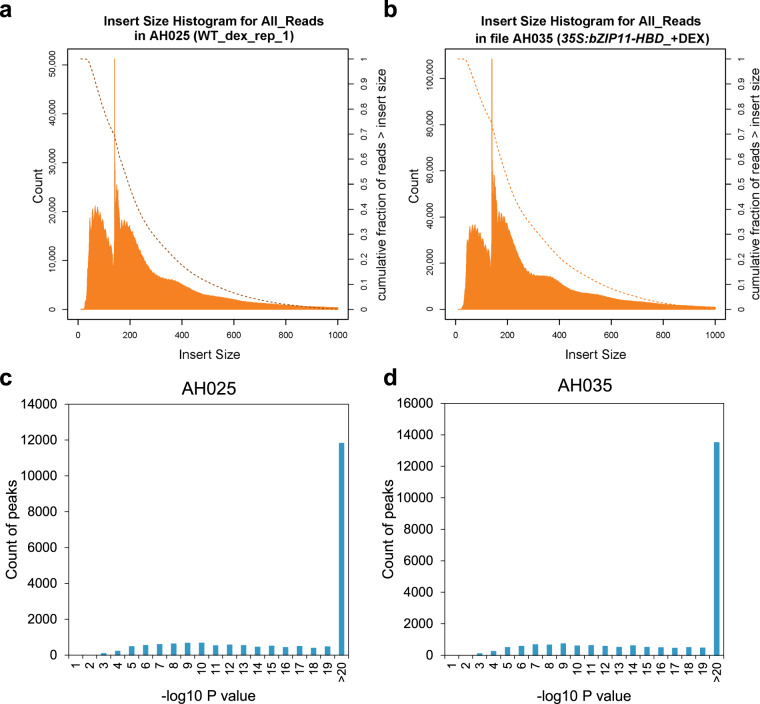
Quality metrics of ATAC-seq reads. (**a,b**) Fragment size distribution of ATAC-seq reads for two representative samples: sample AH025 and AH035 respectively. (**c,d**) Peak score (-log10 (P-value)) distribution for representative samples: sample AH025 (**c**) and AH035 (**d**).

**Fig. 4:**
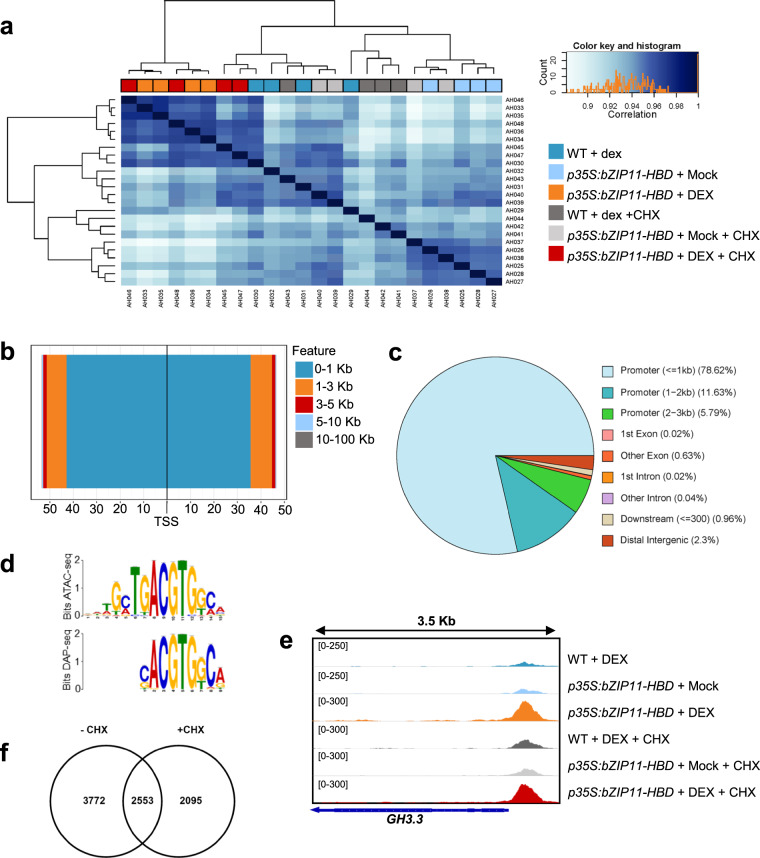
Features of ATAC-seq peaks identified in this dataset. (**a**) Hierarchical clustering of samples based on sample genotype, treatment and replicate number. (**b**) Schematic representation of the distribution of DAR positions upstream and downstream from the TSS of the nearest annotated genes. (**c**) DAR Classification into genic region, based on their location to the nearest gene. (**d**) Motif enrichment analysis obtained in this ATAC-seq (top logo) compared to motif enrichment obtained from DAP-seq data (bottom logo). (**e**) Genome browser views showing chromatin accessibility around *GH3.3* locus in response to bZIP11 in different samples of this experiment. (**f**) Overlap of differentially accessible regions (DARs) determined when bZIP11 induced samples were compared to both wild type (WT) and Mock-treated samples both without cycloheximide (-CHX bubble) and with CHX (-CHX bubble).

We then evaluated the quality and content of the entire ATAC-seq dataset. Most reads were less than 150 bp, consistent with being shorter than one nucleosome (Fig. 3a,b)^34^ and most peaks showed a -log10 P-value score greater than 20, indicating the low likelihood of the peak calling occurring by chance (Fig. 3c,d). Samples where bZIP11 was induced cluster together in a hierarchical ranking plot, depicted by orange (-CHX) and red (+CHX) samples (Fig. 4a). Using differentially accessible peaks, we plotted chromatin accessible signals around genes in response to bZIP11 induction. The regions around transcription start sites are enriched, which is expected for a TF (Fig. 4b). Over 90% of DARs are found in promotor regions of genes, which is expected for a small genome like Arabidopsis (Fig. 4c). In addition, the DAR sequences identified in response to bZIP11 induction, both with and without CHX, were found to be highly enriched in the bZIP CRE, G-box motif (Fig. 4d). This motif closely resembles to the motif identified through DNA Affinity Purification and sequencing (DAP-seq) in response to bZIP11 (Fig. 4d)^9^, supporting that these DARs are regulated by bZIP11. Finally, we tested whether bZIP11 induction efficiently made chromatin more accessible on expected targets. To do so, we visualised the result of the ATAC-seq on *GH3.3* locus, known to be directly regulated at the chromatin level by bZIP11^11^. The results indicate that the chromatin is more accessible when bZIP11 is induced in the *p35S:bZIP11-HBD* samples in absence or presence of CHX (Fig. 4e), consistent with the fact that bZIP11 regulates *GH3.3* at the chromatin level and that the induction of bZIP11 successfully regulated chromatin accessibility in this experiment. Altogether, these data indicate that bZIP11 regulates chromatin to enhance the accessibility of *cis* elements to *trans* regulatory factors, and that this regulatory mechanism concerns a large portion of bZIP11 targets.

To validate direct bZIP11-induced regions of chromatin accessibility, this experiment was repeated both with and without CHX. Without CHX, ATAC-seq determined 10,140 bZIP11-induced DARs, compared to WT, and 11,236 DARs, compared to Mock-treated samples (Table 1). Using ChIPseeker^29^, non CHX DARs were mapped to 6,325 genes (Supplementary Fig. 1a). In the presence of CHX, ATAC-seq determined 8,154 bZIP11-induced DARs, compared to WT, and 11,483 DARs, compared to Mock-treated samples (Table 1). With CHX, DARs were mapped to 4,649 genes (Supplementary Fig. 1b). There are fewer genes made accessible in the presence of CHX, indicating that the treatment worked. Interestingly, in the presence of CHX, there are 3,772 lost genes and 2,095 gained genes (Fig. 4f). This is expected as often multiple proteins are required for both increasing and decreasing chromatin accessibility. To determine which genes are likely to be made accessible directly by bZIP11, the genes corresponding to DARs in CHX-treated and non-treated samples were compared (Fig. 4f). Genes which were annotated in both data sets provide a list of 2,553 genes which are likely to have chromatin accessibility regulated by bZIP11 directly (Supplementary Table 2).

## Supplementary information

Supp. Table S1

Supp. Table S2

Supp. Fig. S1

## Data Availability

All codes used for this study are available on GitHub (https://github.com/AliciaHellens/bZIP11_ATAC-seq).
