# Supplementary material for: Genome-wide identification of accessible chromatin regions by ATAC-seq upon induction of the transcription factor bZIP11 in Arabidopsis
Source: Sci Data. 2023 Jul 27;10:490. doi: 10.1038/s41597-023-02395-6 (PMC10374617; doi:10.1038/s41597-023-02395-6)
Supplement: Supplementary file 1 — Supp. Table S1 [file 41597_2023_2395_MOESM1_ESM.pdf]

Supplementary Table 1: Primers used for qRT-PCR in this study.

Name	Primer sequence	F/R	Gene	Gene ID
qAtActin-F-universal	AGTGCTCGTACAACCGGTATTGT	F		
qAtActin-R1	GATGGCATGAGGAAGAGAGAAAC	R1	*ACT2*	At3g18780
qAtActin-R2	GAGGAAGAGCATACCCCTCGTA	R2	*ACT7*	At5g09810
qAtActin-R3	GAGGATAGCATGTGGAAGTGAGAA	R3	*ACT8*	At1g49240
qAt18S-F	TTCCTAGTAAGCGCGAGTCATCA	F	*18S*	18S rRNA
qAt18S-R	GAACACTTCACCGGATCATTCAAT	R		
qAtTUB-F	TTCACAGCAAGCTTACGGAGGTCA	F	*TUB3*	At5g62700
qAtTUB-R	TGGTGGAGCCTTACAACGCTACTT	R		
qAtASN1-F	CACGCTGCTCAAAATGTCAATG	F	*ASN1*	At3g47340
qAtASN1-R	CAGGAACCGTTAGTCTCGCAGA	R		
