# Supplementary figures and images for: Genome-wide identification of accessible chromatin regions by ATAC-seq upon induction of the transcription factor bZIP11 in Arabidopsis
Source: Sci Data. 2023 Jul 27;10:490. doi: 10.1038/s41597-023-02395-6 (PMC10374617; doi:10.1038/s41597-023-02395-6)

**a**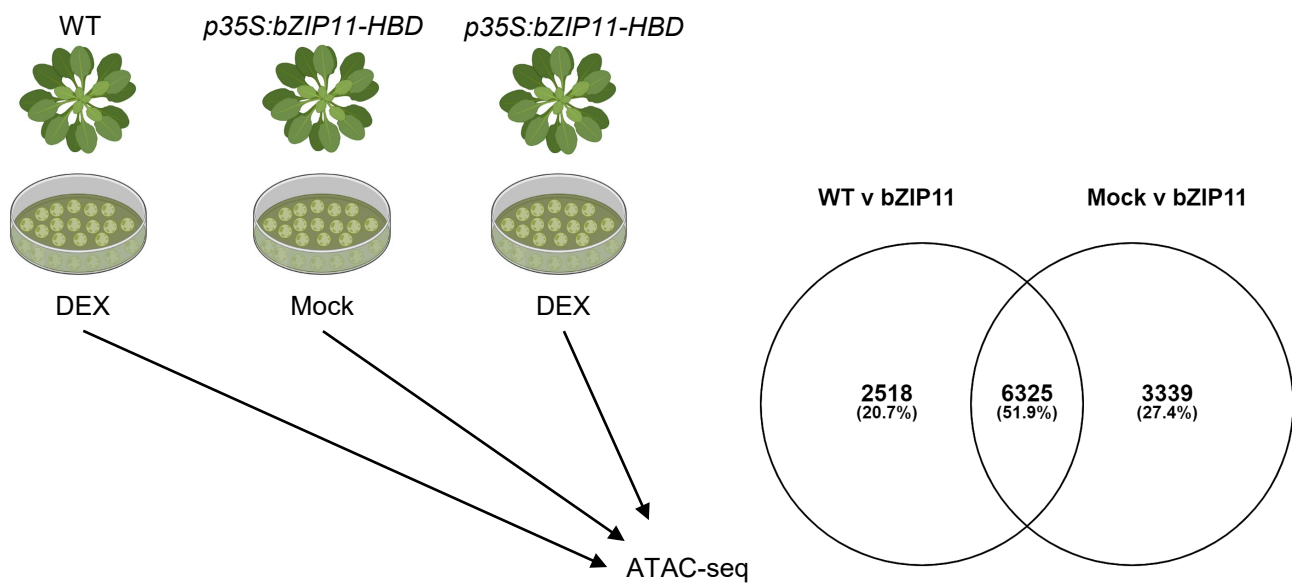**b**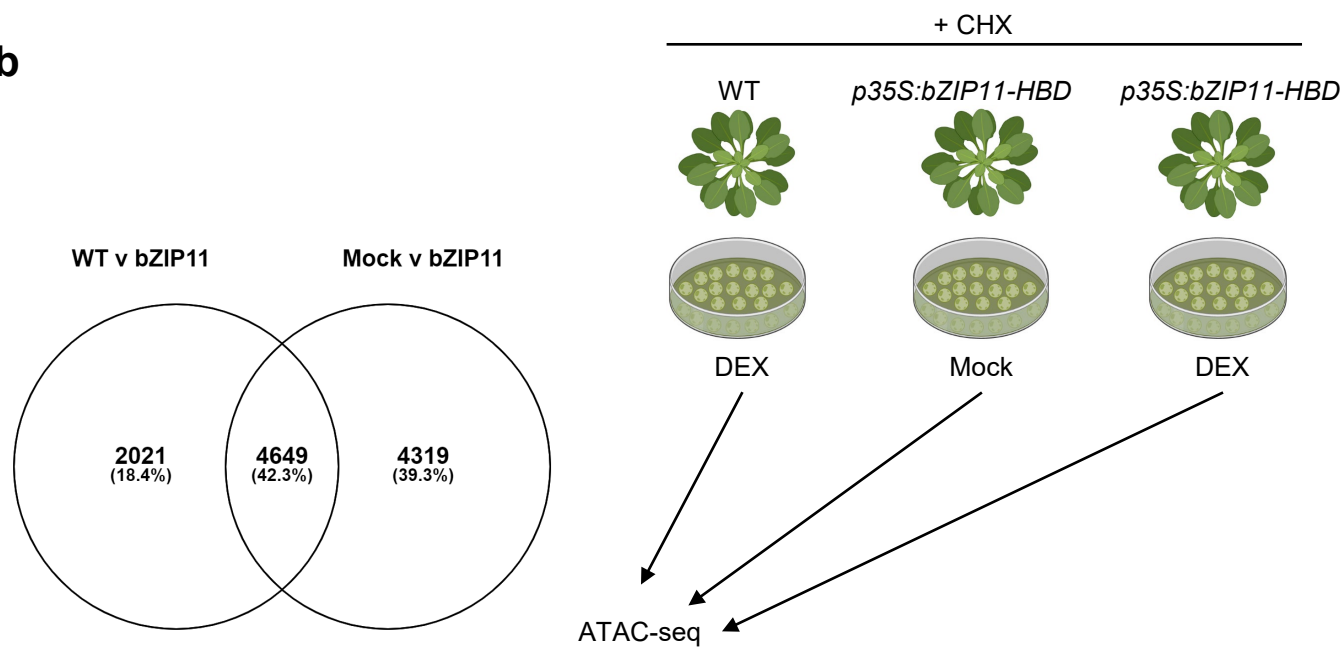

Supplement: Supplementary file 3 — Supp. Fig. S1 [file 41597_2023_2395_MOESM3_ESM.pdf]
